# Four-Year Effects of a Computer-Based Brief Alcohol Intervention Targeting Alcohol Users in the General Population: Randomized Controlled Trial

**DOI:** 10.2196/77921

**Published:** 2025-12-02

**Authors:** Andreas Staudt, Ulrich John, Jennis Freyer-Adam, Gallus Bischof, Maria Zeiser, Sophie Baumann

**Affiliations:** 1 Institute and Policlinic of Occupational and Social Medicine, Faculty of Medicine, Dresden University of Technology, Fetscherstr. 74, Dresden, 01307, Germany, +49 3513177452; 2 Department of Prevention Research and Social Medicine, Institute of Community Medicine, University Medicine Greifswald, Greifswald, Germany; 3 Department of Psychiatry and Psychotherapy, University of Lübeck, Lübeck, Germany; 4 Department of Methods in Community Medicine, Institute of Community Medicine, University Medicine Greifswald, Greifswald, Germany

**Keywords:** alcohol consumption, drinking, brief intervention, screening, proactive, prevention, public health

## Abstract

**Background:**

Brief alcohol interventions aim at motivating individuals to reduce their drinking and encompass a multitude of activities with limited duration or frequency such as counseling or individualized feedback. Brief interventions are effective in alcohol users who exceed the low-risk drinking limits. Considering the health risks associated with alcohol consumption below these limits, brief interventions should be targeted at all alcohol users to maximize their public health impact.

**Objective:**

The study aimed (1) to test the long-term effects of a brief alcohol intervention consisting of computer-generated individualized feedback letters among individuals who consume alcohol, irrespective of how much; and (2) to explore how intervention effects may be moderated by alcohol use severity and school education.

**Methods:**

In the waiting area of the municipal registry office in Greifswald, Mecklenburg-Western Pomerania, Germany, a general population sample of 1646 adults (n=920, 55.89% women; mean age 31.0, SD 10.8 y) who reported alcohol use at least once in the past year were randomized to an intervention (n=815, 49.51%) or a control group (n=831, 50.49%). The intervention comprised up to 3 computer-generated individualized feedback letters based on the transtheoretical model of behavior change at baseline and after 3 and 6 months. The control group received assessment only at the same time points and no feedback. The outcome was a change in self-reported number of drinks per week from baseline to follow-up after 36 and 48 months. Moderators of intervention efficacy were self-reported alcohol use severity (low risk vs at-risk drinking) and school education (<12 y vs ≥12 y) at baseline. Data were analyzed using latent growth modeling with full-information maximum likelihood estimation, ensuring an intention-to-treat analysis. Bayes factors (BFs) were calculated to estimate the sensitivity of evidence.

**Results:**

Unadjusted and adjusted models revealed no group difference after 36 months (incidence rate ratio 1.05, 95% CI 0.87‐1.27; BF=0.37). After 48 months, a decrease in weekly alcohol consumption was observed in the control group and no change in the intervention group (incidence rate ratio 1.29, 95% CI 1.05‐1.57; BF=0.16*),* indicating strong evidence against the hypothesized intervention effect. Intervention efficacy was not moderated by alcohol use severity or school education at baseline.

**Conclusions:**

In a randomized controlled trial, no evidence for the efficacy of individualized feedback letters was found after 3 and 4 years. Unexpectedly, drinking reductions in the control group and no change in the intervention group were observed 4 years after the study start. Intervention strategies effective in at-risk drinkers may necessitate adaptation for applicability to alcohol users as a whole.

## Introduction

Brief alcohol intervention is an umbrella term for a variety of interventions that are characterized by limited intensity or duration, and the common goal of motivating recipients to reduce their alcohol consumption. Examples encompass counseling, advice, and motivational feedback, which can be delivered face-to-face or remotely through digital technologies. There is clear evidence that brief interventions in general can reduce alcohol use in adults who exceed low-risk drinking limits [1-5]. This also applies to interventions that deliver automatically generated, personalized motivational feedback [6-10]. The use of digital technologies such as expert system software, that is, computer programs that automatically evaluate individual information and compile tailored feedback based on a set of predefined rules [11], enables the provision of highly individualized and motivation-enhancing feedback in a reliable and resource-efficient manner [12]. The feedback itself may be delivered web-based [13], through apps [14], short messages [15], letters [9], or a combination thereof [16], whereas the common core is a digitalized expert system.

Crucial questions remain concerning the applicability of brief alcohol interventions such as personalized motivational feedback to the broader population. Low-risk drinking limits [ 17] imply a threshold below which the risk of disease or premature death is marginal and considered acceptable. Therefore, low-risk drinkers were virtually not represented in the brief intervention trials to date.

However, studies emerging in recent years challenge the common practice of excluding them from interventions to reduce alcohol use. These studies revealed that methodological problems (eg, misclassification and uncontrolled confounding) that bias drinking risk estimates downward were common in past research, suggesting that the risk associated with low-risk alcohol intake may be greater than previously estimated [18-21]. Even minimal drinking amounts have been linked to an increased risk of adverse brain and cardiovascular outcomes, as well as prevalent cancers [22-25]. The large proportion of low-risk drinkers in the population might imply high numbers of cases of disease and death below previously recommended thresholds for at-risk drinking [26], which are neither marginal nor acceptable. Therefore, the greatest impact on health can be made by addressing alcohol use in the population as a whole. Accordingly, the latest (inter)national drinking guidelines do not differentiate between low-risk and at-risk alcohol consumption anymore [27-29].

There is a paucity of evidence regarding the long-term effects of brief alcohol interventions. Only very few studies included in the most recently published systematic reviews and meta-analyses investigated treatment effects after more than 1 year [1,3-8]. The impact of low-threshold brief interventions may not only unfold in the weeks directly following the intervention. It may take time before the applied behavior change techniques affect people’s motivation and ultimately, their behavior [7,30,31]. The most influential theories of health behavior change posit that intention is the best predictor of actual change [32-35]. However, the promotion of motivation to change does not guarantee a healthier lifestyle, as is well-documented in the intention-behavior gap [36]. Individuals may reside in a motivational state, contemplating the idea of drinking less alcohol, for a prolonged period of time before an actual change in alcohol consumption manifests itself, or possibly never does. Translated to the prevention context, the effects of brief alcohol interventions may require time to become measurable. To provide an example, reflecting on the pros and cons of drinking, re-evaluating this decisional balance from time to time, eventually setting the goal of drinking less, as well as reaching this goal might be a protracted process [37,38].

The burden of alcohol-related harm is unequally distributed in the general population, with certain groups, such as those with low levels of education, experiencing disproportionately high levels of alcohol-related morbidity and mortality [39-42]. Although the mechanisms underlying this so-called alcohol harm paradox are not well understood yet [43], reducing alcohol-attributable social inequalities is a fundamental public health goal. Alcohol prevention efforts should therefore be systematically evaluated for their equity impact [44], ie, their ability to reduce (positive impact), maintain (neutral impact), or increase (negative impact) health-related social inequalities. On the one hand, lower-educated groups are less likely to accept an offered lifestyle intervention [45,46]. On the other hand, there is considerable evidence that brief interventions are at least as beneficial for alcohol users with low as they are for those with higher socioeconomic status [6,47,48]. Regarding the brief alcohol intervention investigated in this study, evidence after 12 months suggested that alcohol users with lower school education benefited, while those with higher school education did not [49]. An important question is whether this positive equity impact could be sustained over a longer period of time.

In this study, a general population sample of adults who reported any alcohol use in the past year received either computer-generated individualized feedback or assessment only. No clear evidence of efficacy was observed after 1 year [50]. However, the intervention was found to reduce drinking among low-risk drinkers 6 months after enrollment [50]. Given these promising findings after 6 months against the lack of evidence regarding the differential efficacy of brief alcohol interventions in subgroups with different drinking patterns, in particular, those below low-risk drinking limits, we aimed to further explore how intervention effects might differ between low-risk and at-risk drinkers.

The first aim of this study was to compare the change in self-reported alcohol use between the intervention and control groups from baseline to 3 and 4 years after study start. Based on increasing effects of a similar intervention after 2 years in German hospital inpatients [9,51], we expected lower alcohol use in participants randomized to the intervention compared to the control group. The second aim was to explore to what extent the intervention effects, after 3 and 4 years, were moderated by alcohol use severity and school education.

## Methods

### Study Design

This paper reports 4-year outcome data from the extension of the 2-arm randomized controlled trial, “Testing a proactive expert system intervention to prevent and to quit at-risk alcohol use” (PRINT), which was prospectively registered at the German Clinical Trials Register (DRKS00014274, date of registration: March 12, 2018) [52]. The trial protocol can be found in a study by Baumann et al [53]. Primary and secondary outcome data after 12 months have been published elsewhere [50]. The CONSORT-EHEALTH (Consolidated Standards of Reporting Trials of Electronic and Mobile Health Applications and Online Telehealth) guidelines can be found in Checklist 1.

### Participants and Procedure

#### Recruitment and Randomization

The sample was recruited between April 2018 and June 2018 at the municipal registry office in Greifswald, Mecklenburg-Western Pomerania, Germany. Study assistants proactively approached each person who appeared in the waiting area and asked them to participate in a short health survey. The survey questions were answered on tablet computers provided by study staff. Visitors to the registry office had to be aged between 18 and 64 years, cognitively and physically able, and have sufficient language and literacy skills to be eligible. People were also excluded if they had already participated in a previous visit or if they worked at the research institute conducting the study.

The survey included screening questions to determine whether respondents were eligible. Those who reported any alcohol consumption in the past year were given detailed information by the study assistants. Individuals without a permanent address or telephone number were excluded. Those who provided written informed consent were randomized to an intervention or a control group. Participants were randomized by tablet computers in a 1:1 allocation ratio based on a random number table and individuals as units in a simple randomization procedure.

#### Blinding

Participants were blinded to their group assignment during recruitment at the registry office. After receiving the intervention or not, participants were aware whether they belonged to the intervention or control group. Study assistants were blinded to the allocation sequence during randomization and remained blinded to the participants’ group affiliation throughout the trial.

#### Intervention Group

Participants in the intervention group received feedback letters at baseline, at 3 and 6 months, described in more detail in a study by Baumann et al [50]. A translated and annotated example feedback letter can be found in Multimedia Appendix 1. For the intervention group, participants had to provide self-report data on the tablet computers during the recruitment in the registry office (baseline), and via computer-assisted telephone interviews (after 3 and 6 mo). The self-reported data were then processed and evaluated by a computer expert system that constituted the digital core of the intervention. The expert system software contained a comprehensive knowledge base of population data for normative comparisons and a large set of predefined rules that enabled the feedback to be tailored to the participants’ sex and age, their alcohol use risk level, as determined by the Alcohol Use Disorders Identification Test (AUDIT) [54] and AUDIT-C [55], and their motivational stage of change according to the transtheoretical model of behavior change. The language and provision of feedback were based on the principles of motivational interviewing [56]. Once participants entered their data, the expert system automatically compiled individualized feedback letters.

All letters contained recommendations that there is no such thing as risk-free alcohol consumption. They also provided personalized normative feedback on the average amount of alcohol consumed per week and the frequency of heavy episodic drinking (defined as drinking ≥4 alcohol drinks for women and ≥5 for men on a single occasion). The intervention letters for low-risk drinkers (AUDIT-C score <4 for women and <5 for men) aimed at reinforcing participants for their low consumption pattern.

The intervention letters for at-risk drinkers (AUDIT-C score ≥4 for women and ≥5 for men) provided individualized feedback based on the transtheoretical model of behavior change [32]. It covered (1) the participants’ motivational stage of change; (2) their decisional balance, which refers to how they considered the pros and cons of their alcohol use; (3) their self-efficacy, which is their confidence in abstaining from alcohol in different situations; and (4) their engagement in prespecified strategies or behaviors that drive progression through the motivational stages, known as cognitive and behavioral processes of change. In addition, at-risk drinkers received feedback on the risks for negative consequences that may result from their drinking.

The intervention letters for those with probable alcohol use disorder (AUD; AUDIT score ≥20) were similar to those for at-risk drinkers with two exceptions. First, the use of professional treatment was encouraged. Second, they were given feedback about negative consequences that they had already experienced.

The intervention letters at 3 and 6 months were amplified by intra-individual comparisons, indicating how the participants had progressed or changed since the last feedback. To receive the full intervention, trial participants were required to provide self-report data in computer-assisted telephone interviews at 3 and 6 months, respectively. These were used by the expert system software to automatically generate the intervention letters.

#### Control Group

The control group received assessment only, that is, they were asked to provide the same self-report data at baseline, 3, and 6 months as the intervention group. However, the control group did not receive any feedback.

#### Follow-Up Assessments

Follow-up data were collected by study staff after 12 months (April 2019 to June 2019), 36 months (April 2021 to June 2021), and 48 months (April 2022 to June 2022) using standardized, computer-assisted telephone interviews. If the participants could not be reached by telephone after 10 contact attempts, questionnaires were sent by email or mail with up to 2 reminders. To ensure high retention rates, follow-ups were announced beforehand.

### Ethical Considerations

The trial was approved by the ethics committees of University Medicine Greifswald (BB 053/19) and TU Dresden (SR-EK-272062020). All participants provided written informed consent and were compensated with 2 vouchers (each valued at €5 [US $5.8]), one at baseline and one after 12 months. In addition, those who provided long-term follow-up data received up to 2 vouchers (each valued at €5 [US $5.8]) after 36 and 48 months, respectively. After completion of the follow-up interviews after 48 months, the data were deidentified by removing the pseudonym, thus ensuring that the dataset used for statistical analysis did not contain any identifying participant information.

### Measures

#### Outcome

Outcome was a change in the number of drinks per week from baseline to the follow-ups after 36 and 48 months. Participants were asked, “How often did you have an alcoholic drink in the past 30 days?” (never or once or 2‐3 times per wk or ≥ 4 times per wk) and “How many alcoholic drinks did you typically have on a drinking day?” (definition of an alcoholic drink was 0.25‐0.3 l beer, 0.1‐0.15 l wine or sparkling wine, or 4 cl spirits or liquor). To determine the number of alcoholic drinks per week, frequency (drinking days in the past 30 d) was multiplied by the quantity (drinks per drinking day), divided by 4.25 (number of weeks in a month), and rounded down to the nearest integer.

#### Moderators

Alcohol use severity was measured with the AUDIT [54]. Its short form, the AUDIT-C [36] comprising the first 3 AUDIT items, was used to define at-risk drinking. Participants were categorized as at-risk drinkers if women had an AUDIT-C [55] sum score of ≥4, and men of ≥5 [57]. Sum scores <4 (women) and 5 (men) indicated low-risk drinking. Participants who had an AUDIT score of ≥20 were categorized as possible AUD. School education (≤9 or 10-11 or ≥12 y of schooling) was derived from participants’ self-reported highest general educational degree at baseline. Due to a small number of participants with possible AUD (8/1646, 0.49%) and with ≤9 years of school education (101/1646, 6.14%), these participants were merged with at-risk drinkers and those with 10 to 11 years of school education, respectively.

#### Covariates

Sex, age (y), employment status (full-time employed or part-time employed or in education or unemployed or other) were assessed at baseline via self-report. Participants were also asked whether they were currently in a relationship (yes or no), how many portions of fruit and vegetables they ate on a typical day, how much time they spent on moderate-to-vigorous physical activity on a typical weekday and weekend day, respectively, and how many cigarettes they smoked on a typical day.

#### Sample Size Calculation

The sample size calculation for the PRINT trial was based on a hypothesized 15% difference between intervention (expected average of 8.5 alcoholic drinks per week) and control group (expected average of 10 alcoholic drinks per week) at the 12-month follow-up [50], derived from the results of 41 alcohol surveys [58]. The count outcome was expected to follow a negative binomial distribution with a dispersion parameter of 1.0. Based on 80% power, 5% significance level, and 20% drop-out rate from baseline to the 12-month follow-up, the planned sample size was 1648. After receiving renewal funding for long-term follow-ups, 1581 out of 1646 (96.05% of the total sample) participants still had an active consent to be contacted for additional follow-up interviews.

### Statistical Analysis

**Figure 1.**
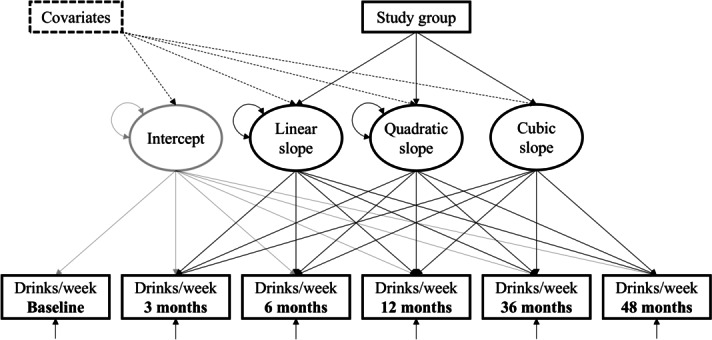
Latent growth model representing the change in number of drinks per week from baseline to 48 months. Ellipses are latent growth factors predicting the repeatedly observed outcome (rectangles) using Poisson regression. Latent growth factors were regressed on study group using linear regression. The model was calculated without and with baseline covariates.

Data were analyzed using Stata (version 18.0; StataCorp LLC) [59] and latent growth curve modeling in Mplus version 8.8 [60]. Following the intention-to-treat principle, the models were calculated including all enrolled participants (N=1646) using full information maximum likelihood estimation with robust SEs. The change in self-reported drinks per week was captured by latent growth factors (Figure 1). Preliminary analyses revealed that negative binomial models as planned in the study protocol [53] did not fit the data due to a dispersion parameter of zero. Therefore, the growth models were calculated using the Poisson distribution. Rescaled likelihood ratio tests indicated that a cubic model with the variance of the cubic factor constrained to zero best represented the growth trajectory over time. Regressing the study group on the latent growth factors allowed us to calculate net changes in drinks per week for the intervention and the control group as well as their difference, expressed as incidence rate ratios (IRR) with 95% CI and *P* values. Bayes factors (BFs) [61] were computed using a BF calculator [62], assuming a half-normal distribution with an expected intervention effect of 15% [53]. BF values below 0.33 were considered as evidence against, values above 3 as evidence for the hypothesized intervention effect, and values in between as data insensitivity [61].

Unadjusted and adjusted growth models were calculated, controlling for baseline covariates that were associated with participation in the follow-up assessments after 36 and 48 months. To this end, 2 logistic regression models were calculated to predict follow-up nonparticipation after 36 and 48 months, respectively (attrition analysis), using baseline information such as sex, age, school education, employment status, relationship status, drinking level, smoking, and study group. In addition, 2 sensitivity analyses are reported in Multimedia Appendix 2. Poisson regression models using multiple imputation to account for missing follow-up data, as well as a pattern mixture model to consider that data might be missing not at random (MNAR).

Finally, it was tested whether intervention efficacy was moderated by alcohol use severity or school education at baseline. Interaction terms between study group and alcohol risk level, and between study group and school education were introduced into the model. Interaction effects are reported as IRRs with 95% CIs. Moderation analyses were adjusted for sociodemographic characteristics (sex, age, employment, and relationship status) and health behaviors (fruit and vegetable intake, physical activity, and smoking) at baseline.

## Results

### Sample Characteristics

**Figure 2.**
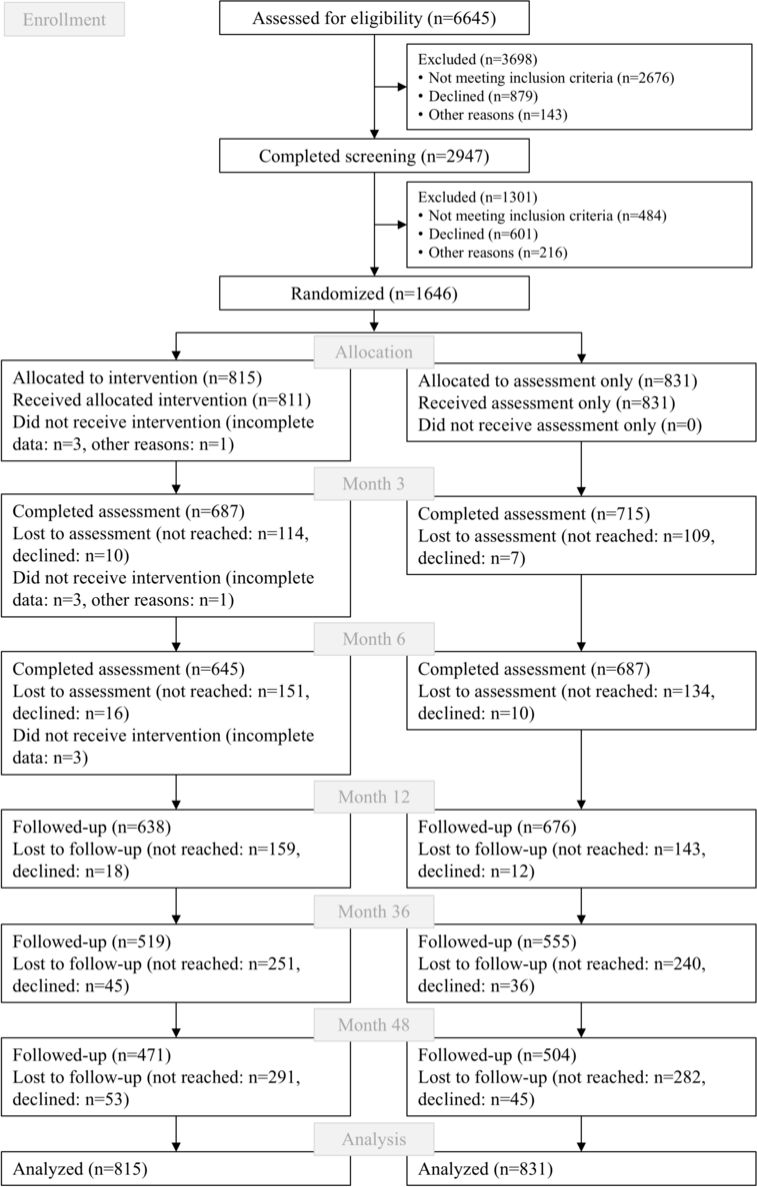
Flow of participants.

Of the 2462 eligible persons in the registry office waiting area, 1646 (66.86%) participated in the trial (Figure 2). The sample (920/1646, 55.89% women) had a mean age of 31.0 (SD 10.8) years. The majority had ≥12 years of school education (1072/1646, 65.13%) and were full-time employed (689/1646, 41.86%) or part-time employed (358/1646, 21.75%). According to the AUDIT-C, 1085 (65.92%) participants reported low risk and 553 (33.59%) at-risk drinking at baseline (Table 1). As reported in a study by Enders et al [63], trial participants were more likely to be younger and report low-risk drinking compared to nonparticipants.

**Table 1.** Sample characteristics (N=1646).

Characteristic	Total sample	Intervention (n=815)	Control (n=831)
Age (y), mean (SD)	31.0 (10.8)	31.2 (10.9)	30.8 (10.8)
Gender (woman), n (%)	920 (55.89)	460 (56.4)	460 (55.4)
School education (y), n (%)			
≤9	101 (6.14)	52 (6.4)	49 (5.9)
10-11	473 (28.74)	248 (30.4)	225 (27.1)
≥12	1072 (65.13)	515 (63.2)	557 (67)
Employment status, n (%)			
Full-time employed	689 (41.86)	343 (42.1)	346 (41.6)
Part-time employed	358 (21.75)	179 (22)	179 (21.5)
In education	444 (26.97)	219 (26.9)	225 (27.1)
Unemployed	53 (3.22)	26 (3.2)	27 (3.3)
Others	102 (6.20)	48 (5.9)	54 (6.5)
In a relationship (yes), n (%)	1044 (63.43)	529 (64.9)	515 (62)
Fruit and vegetable (portions per day), mean (SD)	2.2 (1.5)	2.2 (1.4)	2.2 (1.5)
MVPA^a^ per day (min), mean (SD)	74.4 (83.1)	74.1 (86.4)	74.8 (79.7)
Cigarettes per day, mean (SD)	3.0 (6.2)	2.9 (6.2)	3.1 (6.2)
Alcohol use severity at baseline, n (%)			
Low-risk drinking	1085 (65.92)	545 (66.9)	540 (65)
At-risk drinking	553 (33.60)	267 (32.8)	286 (34.4)
Possible alcohol use disorder	8 (0.49)	3 (0.4)	5 (0.6)
Alcoholic drinks per week (last 30 d), mean (SD)			
Baseline	1.8 (3.9)	1.8 (3.6)	1.8 (4.2)
At 12 months (n=1314)	2.2 (4.7)	2.1 (4.2)	2.3 (5.2)
At 36 months (n=1074)	2.3 (4.7)	2.3 (4.4)	2.3 (5.0)
At 48 months (n=975)	2.2 (4.2)	2.3 (4.5)	2.1 (4.0)

a MVPA: moderate-to-vigorous physical activity.

### Intervention Adherence

Out of 815 participants in the intervention group, 615 (75.5%) received the complete intervention consisting of 3 individualized feedback letters; 103 (12.6%) participants received 2, and 94 (11.5%) received 1 of 3 intervention letters, respectively. The primary reason for nonadherence was that participants could not be reached for the assessments, on which the intervention letters were based.

### Follow-Up Participation

Participation for the follow-up assessments at 36 and 48 months was 65.2% (1074/1646) and 59.2% (975/1646), respectively (Figure 2). Nonparticipation in the follow-up at 36 months was associated with younger age (odds ratio [OR] 0.97, 95% CI 0.96‐0.98; *P*<.001) and a lower level of school education (OR 0.34, 95% CI 0.26‐0.44; *P*<.001). Baseline smokers (OR 2.08, 95% CI 1.62‐2.67; *P*<.001) and at-risk drinkers (OR 1.33, 95% CI 1.04‐1.70; *P*=.024) were more likely to drop out at 36 months. Nonparticipation at 48 months was also associated with younger age (OR 0.97, 95% CI 0.96‐0.98; *P*<.001), a lower level of school education (OR 0.32, 95% CI 0.25‐0.42; *P*<.001), and not being in a relationship at baseline (OR 0.75, 95% CI 0.59‐0.95; *P*=.016). Baseline smokers (OR 2.06, 95% CI 1.61‐2.63; *P*<.001) and participants who were unemployed compared to full-time employed (OR 2.27, 95% CI 1.15‐4.48; *P*=.018) were more likely to drop out at 48 months. At-risk drinkers at baseline also tended to be more likely to drop out at 48 months, but this was not statistically significant (OR 1.18, 95% CI 0.93‐1.50; *P*=.180).

### Intervention Efficacy

No group differences were found regarding the change in alcoholic drinks per week from baseline to 36 months (Table 2). BFs tended to indicate evidence for the null, that is, no difference between groups, but remained in the range where the data are to be considered insensitive. At 48 months, the unadjusted growth model revealed a decrease in weekly alcohol consumption in the control group and no change in the intervention group. This difference was statistically significant (IRR 1.29, 95% CI 1.05‐1.57; *P*=.015) and remained after adjustment for baseline covariates (IRR 1.27, 95% CI 1.06‐1.54; *P*=.012*).* BFs indicated strong evidence against the hypothesized intervention effect.

**Table 2.** Model-implied between-group differences (N=1646)^e^.

	Unadjusted model		Adjusted model^b^	
	IRR^f^ (95% CI)	BF*^d^*	IRR (95% CI)	BF*^d^*
At 36 months	1.05 (0.87‐1.27)	0.37	1.06 (0.89‐1.28)	0.35
At 48 months	1.29 (1.05‐1.57)	0.16	1.27 (1.06‐1.54)	0.17

a Cubic latent growth models for Poisson-distributed data. Outcome was net change in the number of alcoholic drinks per week since baseline.

b Adjusted for baseline covariates sex, age, education, employment, smoking, relationship status, and alcohol use severity.

c IRR: incidence rate ratio.

d BF: Bayes factor which was calculated using a half-normal distribution with an expected intervention effect of 15%*.*

### Sensitivity Analyses

The results of the Poisson regression models using multiply imputed outcome data were consistent with the results of the latent growth models (Multimedia Appendix 2). The results of the pattern mixture models were associated with a high degree of uncertainty and suggested no group differences at 36 and 48 months (Multimedia Appendix 2).

### Moderation Analyses

There were no group differences between low-risk and at-risk drinkers in the change in alcoholic drinks per week from baseline to 36 and 48 months (Table 3; IRR 0.94, 95% CI 0.66‐1.35 and IRR 0.85, 95% CI 0.59‐1.23, respectively). There were also no group differences between participants with <12 years and those with ≥12 years of school education in the change in alcoholic drinks per week from baseline to 36 and 48 months (Table 4; IRR 0.73, 95% CI 0.47‐1.12 and IRR 0.76, 95% CI 0.48‐1.20*,* respectively). Thus, intervention efficacy was not moderated by alcohol-related risk level or school education at baseline.

**Table 3.** Intervention effects by alcohol-related risk level at baseline (N=1646)^a^.

	Difference between intervention and control group		Interaction effect, IRR^b^ (95% CI)
	Low-risk drinkers, IRR (95% CI)	At-risk drinkers, IRR (95% CI)	
At 36 months	1.04 (0.81‐1.33)	1.10 (0.85‐1.43)	0.94 (0.66‐1.35)
At 48 months	1.18 (0.93‐1.51)	1.39 (1.06‐1.79)	0.85 (0.59‐1.23)

a Cubic latent growth model for Poisson-distributed data, adjusted for baseline covariates sex, age, school education, employment, relationship status, smoking, physical activity, and fruit and vegetable intake. Outcome was net change in the number of alcoholic drinks per week since baseline.

b IRR: incidence rate ratio.

**Table 4.** Intervention effects by school education (N=1646)^a^.

	Difference between intervention and control group		Interaction effect, IRR^b^ (95% CI)
	<12 years of school education, IRR (95% CI)	≥12 years of school education, IRR (95% CI)	
At 36 months	0.84 (0.58‐1.22)	1.15 (0.94‐1.42)	0.73 (0.47‐1.12)
At 48 months	1.04 (0.70‐1.56)	1.37 (1.11‐1.70)	0.76 (0.48‐1.20)

a Cubic latent growth model for Poisson-distributed data, adjusted for baseline covariates sex, age, employment, relationship status, smoking, alcohol use, physical activity, and fruit and vegetable intake. Outcome was net change in the number of alcoholic drinks per week since baseline.

b IRR: incidence rate ratio.

## Discussion

The data of this randomized controlled trial using a general population sample of alcohol users showed no evidence of effects of individualized feedback based on a computer expert system after 4 years. Unexpectedly, we observed drinking reductions in the control group and no change in the intervention group 4 years after study start. Long-term intervention effects were not moderated by alcohol use severity or school education.

It is puzzling and somewhat paradoxical that individualized feedback might have produced potentially negative long-term effects, considering that (1) there was no difference between study groups after 1 [50] and 3 years; (2) the intervention was designed according to the principles of motivational interviewing [56], using benevolent and appreciative language to address the participants’ motivation and beliefs about their alcohol use; (3) preventive intervention are usually assumed to have few risks; and (4) evidence on the existence of adverse and unintended effects of brief alcohol interventions is scarce [64].

Among the studies included in the meta-analyses and reviews published in the last years [1,3,4,6,65-68], only 1 trial was identified that reported potentially adverse effects of a brief intervention targeting alcohol use [69]. After 3 and 6 months, the proportion of high-risk drinkers in a sample of patients with tuberculosis in South Africa decreased more strongly in the control group (treatment as usual) than in the brief counseling intervention group [69]. However, comparability appears to be very limited due to differences in study characteristics. The almost complete lack of studies reporting unintended brief intervention effects may be due to publication bias or to the fact that brief interventions usually operate at such a low motivational threshold that adverse effects seem unlikely. This makes it all the more important to disentangle the long-term effects mentioned earlier.

There are at least 3 possible explanations for our findings. First, the feedback provided in the intervention letters may not have had the desired effect, or even an opposite effect for some participants. Two-thirds of the sample were low-risk drinkers, a group that has been largely absent from brief intervention trials to date. The feedback may have been insufficiently tailored to the needs of this group. The motivational processes involved in reducing alcohol consumption may differ for different subgroups of drinkers. For instance, personalized normative feedback assumes that people underestimate their drinking in relation to their peers [67]. This may not be the case for all members of our population-based sample, which included a multitude of drinking patterns. Rather than enhancing the motivation to drink less, the feedback may have provided some participants with a form of justification for their drinking. If that is the case, future interventions may better omit risk levels such as low-risk or at-risk drinking that are no longer supported by evidence [18-25]. Second, participation in the follow-up assessment after 4 years was the lowest in the trial (59%) and selective. Although our analysis strategy can deal with data missing at random [70], the possibility of systematic bias due to data not missing at random can never be completely ruled out [71]. Incorporating nonignorable missingness into latent growth models is based on untestable assumptions and can result in a high degree of uncertainty [72] as was the case in our sensitivity analyses (Multimedia Appendix 2). Third, the follow-up after 4 years was conducted between April 2022 and June 2022, a period during which the German general population had experienced 2 years of restrictions and constantly shifting life circumstances due to the COVID-19 pandemic. The pandemic affected people’s alcohol consumption [73,74], their health literacy [75], as well as their trust in science [76]. During the follow-up interviews, some participants spontaneously indicated that they had retained the feedback letters and re-read them from time to time. Although we were unable to investigate this more rigorously, participants in the intervention group might have re-evaluated their feedback during the COVID-19 pandemic. Albeit highly speculative, it is possible that this could have facilitated undesired effects through an unintended false sense of reassurance or influenced the accuracy of self-reported drinking in the intervention group but not in the control group.

Temporary positive intervention effects among low-risk drinkers [50] and participants with a low or medium educational background [49] were not sustained over the longer term. In a high consumption country such as Germany [77], where the social pressure to drink is high, individualized feedback might need to be provided more frequently and consistently to achieve prolonged drinking reductions.

The public health potential of computer expert systems as brief intervention tools lies in their capacity to reliably provide highly individualized feedback to large groups of alcohol consumers, with relatively low effort once the expert system has been developed. This is particularly relevant given that brief alcohol interventions should not be limited to individuals who exceed former low-risk drinking limits, considering the health risks associated with drinking patterns below these limits [18-24]. Consequently, the target group of behavior-directed alcohol prevention encompasses a substantially larger share of the population than before. Motivational feedback based on digitalized expert systems is suitable for this purpose due to its efficiency and translational potential. In an increasingly digitalized world, the possibilities for providing visually appealing and easily accessible expert system feedback are manifold and growing constantly. In contrast, more terrestrial ways of feedback delivery, such as written letters sent via postal mail as used in the present study, may become more salient, leaving a lasting impression in a world characterized by mostly digital ways of communicating. Future studies could explore the role of delivery mode on how motivational feedback is processed and evaluated by recipients.

This study represents one of the first attempts to incorporate new evidence regarding the risk from low drinking amounts [18-21] into a universal preventive intervention targeting all people who drink alcohol, regardless of how much. High participation and retention rates were achieved with a large population-based sample, which was comparable to the general population regarding the prevalence of problematic alcohol use [78]. The intention-to-treat analysis, including several sensitivity analyses, ensures the reliability of the conclusions drawn from the study data. However, the following limitations need to be acknowledged. First, the participation was selective, potentially introducing bias through nonignorable missingness. Second, alcohol consumption was self-reported using a quantity-frequency approach, which is known to be subject to underreporting by respondents [79]. Third, the assessment of the primary outcome may not have been sensitive and fine-grained enough to detect changes among participants with infrequent drinking patterns. Fourth, the PRINT trial was originally designed to investigate intervention effects after one year and the required sample size was calculated based on group differences within this period of time. Following the acquisition of renewal funding, we were able to explore long-term intervention and moderation effects. Since the long-term follow-ups after 3 and 4 years were not initially scheduled in the PRINT trial, the results should be considered as exploratory in nature, with potentially limited statistical power.

This study contributes to the literature on brief interventions by providing insight into the long-term effects of computer-generated individualized feedback letters. While previous studies have largely focused on individuals who consumed alcohol above a certain risk threshold, this study expands the scope to include all drinkers.

## Supplementary material

10.2196/77921 Multimedia Appendix 1 Translated and annotated example intervention letter.

10.2196/77921 Multimedia Appendix 2 Sensitivity analyses.

10.2196/77921 Checklist 1 CONSORT-EHEALTH checklist.

## Abbreviations

AUD: alcohol use disorder
AUDIT: Alcohol Use Disorders Identification Test
AUDIT-C: Alcohol Use Disorders Identification Test–Consumption
BF: Bayes factor
CONSORT-EHEALTH: Consolidated Standards of Reporting Trials of Electronic and Mobile Health Applications and Online Telehealth
IRR: incidence rate ratio
OR: odds ratio
