# Supplementary material for: Four-Year Effects of a Computer-Based Brief Alcohol Intervention Targeting Alcohol Users in the General Population: Randomized Controlled Trial
Source: J Med Internet Res. 2025 Dec 2;27:e77921. doi: 10.2196/77921 (PMC12671907; doi:10.2196/77921)
Supplement: Multimedia Appendix 1 [file jmir-v27-e77921-s001.pdf]

print

Universitätsmedizin GREIFSWALD

Institute of Social Medicine and Prevention
Director: Prof. Dr. U. John

University of Greifswald / Institute of Social Medicine and Prevention
Walther-Rathenau-Str. 48 / 17475 Greifswald / PRINT

Mr.
Test Person
123 Street
Germany

Greifswald, 16 May 2018

Your personal PRINT feedback

Dear Mr. Person,

Thank you for taking part in in the PRINT study. You will now receive personal feedback based on the information you provided.

Where do you stand?

You think about your alcohol consumption from time to time. But you are not yet fully convinced that you want to change it.

Summarizing feedback:
- Feedback of **alcohol use risk status** (low-risk, at-risk, or AUD likely) + change since last letter (if applicable)
- Feedback of **motivational status** (precontemplation, contemplation, preparation, or action) + change since last letter (if applicable)

What is low-risk alcohol consumption?

Most people do not have to give up alcohol completely. The limits of low-risk alcohol consumption are:

Healthy men are recommended to drink…

- …a maximum of 14 alcoholic drinks per week…
- …no more than 4 alcoholic drinks on any one occasion.

Low-risk does not mean *risk-free*. The consumption of alcoholic beverages is generally associated with an increased risk of illness. However, of you stick to the recommended limits, your risk is low. At least in some situations you should avoid alcohol altogether: if you are actively involved in road traffic, are taking medication or are ill. In this way, you are consciously taking responsibility for yourself and others.

What is an **alcoholic drink**?

1 glass of beer (0.25 – 0.3 l)

1 glass of wine or sparkling wine (0.1 – 0.15 l)

4 cl spirits

Low-risk drinking guidelines:
- Gender-specific thresholds
- Graphical information about the size of an alcoholic standard drink
- Note that “low-risk” does not equal no risk for consequences
- Limited validity of guidelines for certain situations (e.g. driving)

A comparison of your average weekly drinking amount

In the following chart you can see, how much you drink compared to others.

Out of 100 men in your age group...

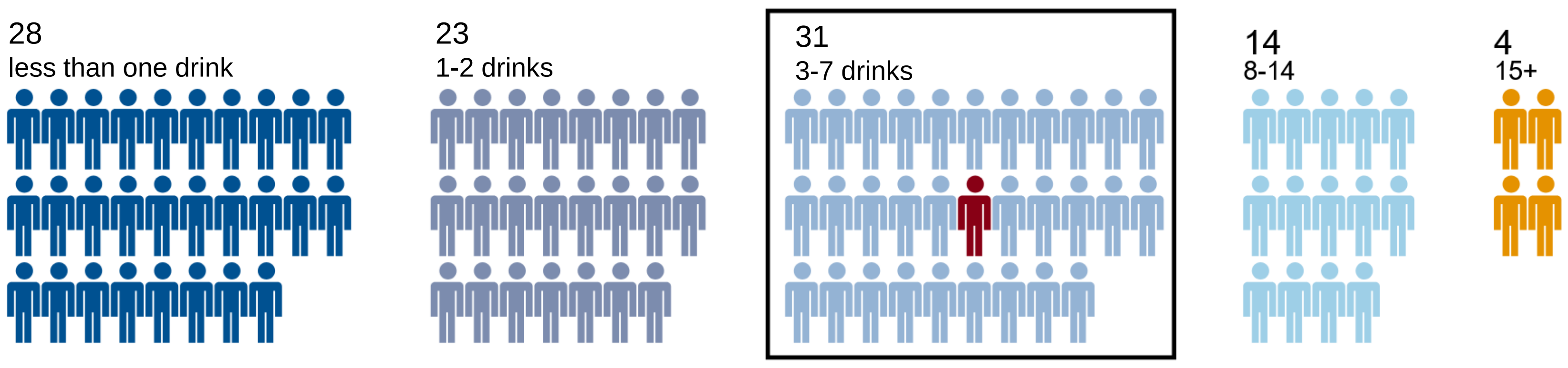

You drink 3-7 alcoholic drinks per week, more than 51% of men in your age group.

Personalized normative feedback on weekly amount of alcohol use:

- Gender- and age-specific
- Graphical and textual display of much alcohol a person drinks per week in comparison to his or her reference group (data from a survey representative of the German general population)

+ ipsative feedback (change since last letter) if applicable

Assessing your personal risk

The amount of alcohol you drink has a direct effect on the risk of negative consequences. These consequences can be of a health-related, psychological, and social nature (e.g. high blood pressure, sleep disorders, strain on interpersonal relationships).

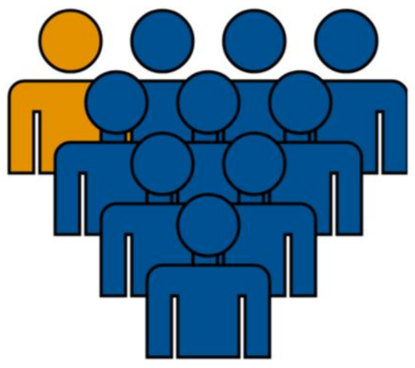

For every 10 persons who drink the amount you indicated, one person must expect negative consequences.

Normative feedback on the risk for consequences associated with the current amount of alcohol use:

- Information on potential negative consequences (e.g. sleep disorders)
- Personalized absolute risk: “One out of ten persons with your current amount of alcohol use must expect negative consequences.”

+ ipsative feedback (change since last letter) if applicable

The amount you drink on one occasion

In the following chart, you can see how often you drink more than 4 alcoholic drinks on a single occasion compared to others.

Drinking more than 4 alcoholic drinks on one occasion report...

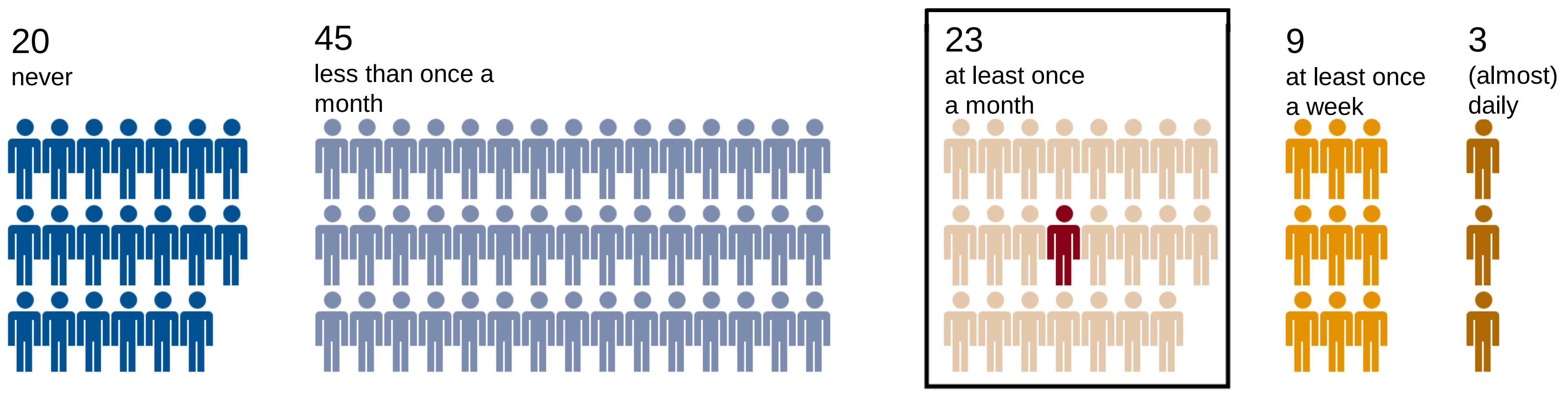

You drink more than 4 alcoholic drinks on one occasion at least once a month, more often than 65% of men in your age group.

Personalized normative feedback on the frequency of heavy episodic drinking:

- Gender- and age-specific
- Graphical and textual display of much alcohol a person drinks per week in comparison to his or her reference group (data from a survey representative of the German general population)

+ ipsative feedback (change since last letter) if applicable

You see more unpleasant than pleasant aspects of alcohol consumption.

What you appreciate about drinking alcohol: It helps you to relax.

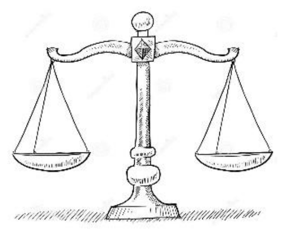

It is good that you consider the following disadvantages of at-risk alcohol consumption into your decision when you think about drinking alcohol or not:

- You may gain body weight.
- You may set a bad example for others.
- You may damage your health.

Feedback on **decisional balance**
- Relative importance of pros and cons of alcohol use
- Highlighting of personally important pros and cons

Compared to other people...

...the unpleasant aspects seem important to you. This critical view is really commendable!

Normative feedback on the relative importance of the unpleasant aspects of alcohol use (cons):
- Comparison with reference group
- Encouraging recipient to reflect on potentially negative aspects

+ ipsative feedback (change since last letter) if applicable

And where do you go from here?

The following recommendations have been proven to be helpful in many cases, similar to your personal situation. They may help you achieve the goal of cutting down drinking. It's up to you to decide whether there are suitable suggestions for you.

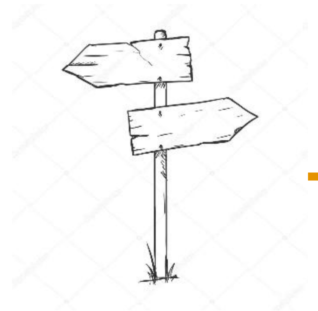

Feedback on **processes of change**:
- Choice of processes tailored to the recipient's motivational status
- Including normative and ipsative feedback (if applicable)

Think about the possible consequences.
You are definitely affected when you hear about the negative consequences of drinking alcohol. This might help you weigh the advantages and disadvantages of low-risk consumption.

Dramatic relief

Pay attention to possible negative consequences for those around you.
You tend not to think much about the effects your level of drinking may have on others. Perhaps there is no reason for you to do so. For some people, it can be useful to picture the situation of those close to them. Has anyone ever expressed concern about the amount of alcohol you drink?

Environmental reevaluation

Be open to new information.
Compared to others, you take information about possible negative aspects of drinking alcohol seriously. This can help you move forward and weigh up the pros and cons.

Consciousness raising

How does your self-image change when you drink?
It can be useful to consider the extent to which drinking alcohol does not match your self-image in certain aspects. For example, have you ever been annoyed by a hangover after a party and not been able to spend the following day as planned?

Self-reevaluation

Attention: Critical situations!

Everyone likes to have an alcoholic drink in certain situations. These make it difficult to change your drinking habits. It is therefore important to think about how to react appropriately in such situations before they happen.

Feedback on **self-efficacy**:
- Information on the importance of personally relevant situations
- Normative feedback on the overall amount of self-efficacy

+ ipsative feedback (change since last letter) if applicable

You have a comparatively high level of confidence in your ability to stick to your goals even in critical situations. This is a good basis if you are planning to change something about your alcohol consumption.

You find it particularly difficult to drink little or no alcohol when you are in a good mood and in social situations. The following suggestions might be helpful:

- Avoid situations, in which a lot of alcohol is consumed.
- Think about the maximum number of drinks you would like to drink beforehand.
- Clearly reject offers to drink alcohol.

Personally relevant situations with particularly low self-efficacy

+ potential coping strategies

Stick with it!

You have already made a big step: You are thinking about drinking less alcohol. We hope to have given you some interesting food for thoughts that you can use for your health and well-being. Keep the letter in a safe place so that you can read it again in the near future. Perhaps it will give you additional impetus to prepare for your goal.

We wish you, Mr. Test Person, all the best!

Concluding remarks:
- Tailored to the recipient's motivational status
- Encouraging recipient to continue effort to consume alcohol on low levels
- Highlighting of benefit for recipient's health

Yours sincerely

The PRINT project team
