# Supplementary material for: Four-Year Effects of a Computer-Based Brief Alcohol Intervention Targeting Alcohol Users in the General Population: Randomized Controlled Trial
Source: J Med Internet Res. 2025 Dec 2;27:e77921. doi: 10.2196/77921 (PMC12671907; doi:10.2196/77921)
Supplement: Multimedia Appendix 2 [file jmir-v27-e77921-s002.pdf]

Multimedia Appendix – Sensitivity analyses

This is a Multimedia Appendix to a full manuscript published in the J Med Internet Res. For full copyright and citation information see http://dx.doi.org/10.2196/jmir.xxxx.

1 Poisson regression models

1.1 Statistical analysis

Full information maximum likelihood estimation as reported in the main analysis has been shown to perform as well as multiple imputation when data are missing at random [1]. However, results could still be sensitive to the method used to account for missing data. Therefore, we generated 100 data sets after a sequence of 100 Monte Carlo Markov chain iterations to impute the number of alcoholic drinks per week at 36 (34.8% missing) and 48 months (40.8% missing). The variables used for the imputation were sex, age, school education, employment, relationship status, smoking, fruit and vegetable intake, physical activity (all baseline), and number of alcoholic drinks per week at 12 months. The imputed datasets were then analyzed using Rubin's rules [2].

To estimate intervention efficacy, unadjusted and adjusted Poisson regression models were calculated. The outcome was the number of alcoholic drinks per week at 36 and 48 months, respectively. Study group (0 = control / 1 = intervention group) was entered as a predictor, while baseline drinking was controlled for.

1.2 Results

No group differences were found at 36 months (upper part of Table S1). At 48 months (lower part of Table S1), the unadjusted model suggested higher weekly alcohol consumption in the intervention group compared with the control group (*IRR* = 1.12; *95% CI* = 1.03-1.21; *P* = .007). This result remained after adjustment for baseline covariates (*IRR* = 1.13; *95% CI* = 1.04-1.22; *P* = .004). According to the Bayes factors, all Poisson regression models provided strong evidence against the hypothesized intervention effect.

Table S1. Poisson regression model results predicting number of alcoholic drinks per week at 36 and 48 months, controlled for baseline drinking

	Unadjusted model			Adjusted model[a]		
	IRR	*95% CI*	*BF*[b]	*IRR*	*95% CI*	*BF*[b]
At 36 months						
Alcoholic drinks / week (baseline)	1.06	1.06-1.07		1.03	1.02-1.04	
Study group (1 = intervention)	1.02	0.94-1.10	0.16	1.03	0.96-1.11	0.14
At 48 months						
Alcoholic drinks / week (baseline)	1.06	1.05-1.06		1.02	1.02-1.03	
Study group (1 = intervention)	1.12	1.03-1.21	0.06	1.13	1.04-1.22	0.05

N = 1,646. Missing outcome data was accounted for by multiple imputation. *IRR* = Incidence rate ratio. *CI* = Confidence interval. *BF* = Bayes factor.

[a] Adjusted for baseline covariates sex, age, education, employment, smoking, relationship status and alcohol use severity.

[b] *BFs* were calculated using a half-normal distribution with an expected intervention effect of 15%.

2 Pattern mixture model

2.1 Statistical analysis

Since there is no way to determine with certainty whether missing follow-up data are missing at random or missing not at random, sensitivity analyses are recommended [3,4]. One possibility in latent growth curve modeling is the pattern mixture approach [5]. For this purpose, the sample was divided into subgroups based on similarities in their patterns of missing data and observed trajectories in the number of alcoholic drinks per week from baseline to 48 months (Figure S1). These three subgroups were 872 (53.0%) complete cases (pattern 1), 286 (17.4%) participants with intermittent missing values who provided follow-up data at least once (patterns 2 to 6), and 488 (29.6%) participants who dropped out of the study before the follow-up assessment at 36 months (patterns 7 and 8).

Multiple group analysis in Mplus was used for the pattern mixture approach. The growth model described in the main paper was estimated separately in each subgroup using the KNOWNCLASS option. Thus, the pattern mixture approach allows the estimation of different growth trajectories in the predefined subgroups. To ensure model identification, inestimable parameters were constrained to the average of the same parameter in the other two available subgroups [6]. Parameter estimates for the total sample were obtained by calculating the weighted average of the group-specific estimates.

2.2 Results

The pattern mixture models showed that both the intervention and control groups tended to reduce their number of alcoholic drinks per week from baseline to 36 and 48 months, respectively. However, there were no group differences at 36 and 48 months (Table S2). It must be acknowledged that these estimates are highly uncertain, given the width of the confidence intervals and the Bayes factors signaling complete data insensitivity.

Table S2. Pattern mixture model between group differences

	Unadjusted model			Adjusted model[a]		
	IRR	*95% CI*	*BF*[b]	*IRR*	*95% CI*	*BF*[b]
At 36 months	1.04	0.13-8.26	0.98	1.11	0.15-8.23	0.98
At 48 months	1.12	0.02-68.79	0.99	1.24	0.02-65.65	0.99

N = 1,646. Cubic latent growth pattern mixture models for Poisson-distributed data. Outcome was net change in the number of alcoholic drinks per week since baseline. *IRR* = Incidence rate ratio. *CI* = Confidence interval. *BF* = Bayes factor.

[a] Adjusted for baseline covariates sex, age, education, employment, smoking, relationship status and alcohol use severity.

[b] *BFs* were calculated using a half-normal distribution with an expected intervention effect of 15%.

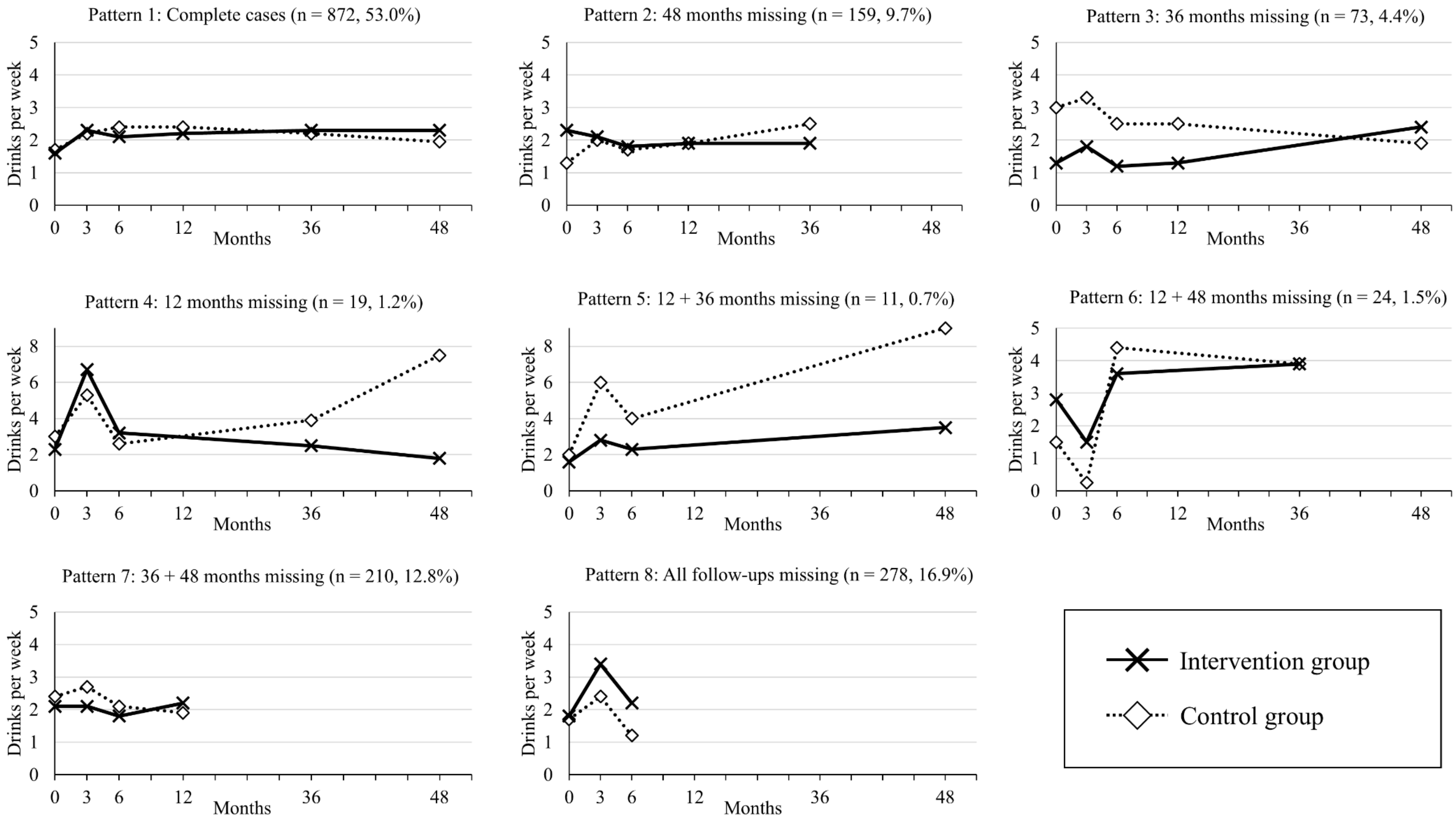

Figure S1. Missing data patterns and observed trajectories in the number of alcoholic drinks per week from baseline to 48 months

References

1. Hallgren KA, Witkiewitz K. Missing Data in Alcohol Clinical Trials: A Comparison of Methods. Alcohol Clin Exp Res 2013;37(12):2152–2160. doi: 10.1111/acer.12205

2. Rubin DB. Multiple Imputation for Nonresponse in Surveys. New York City: J. Wiley & Sons; 1987. doi: 10.1002/9780470316696ISBN:9780471087052

3. Morris TP, Kahan BC, White IR. Choosing sensitivity analyses for randomised trials: principles. BMC Med Res Methodol 2014;14(1):11. doi: 10.1186/1471-2288-14-11

4. National Research Council (US) on Handling Missing Data in Clinical Trials. The prevention and treatment of missing data in clinical trials. Washington (DC): National Academies Press (US); 2010.

5. Enders CK. Missing not at random models for latent growth curve analyses. Psychol Methods 2011;16(1):1–16. doi: 10.1037/a0022640

6. Staudt A, Freyer-Adam J, Ittermann T, Meyer C, Bischof G, John U, Baumann S. Sensitivity analyses for data missing at random versus missing not at random using latent growth modelling: a practical guide for randomised controlled trials. BMC Med Res Methodol 2022;22(1):250. doi: 10.1186/s12874-022-01727-1
